# Respiratory function monitoring during neonatal resuscitation: A systematic review

**DOI:** 10.1016/j.resplu.2022.100327

**Published:** 2022-11-17

**Authors:** Janene H. Fuerch, Marta Thio, Louis P. Halamek, Helen G. Liley, Myra H. Wyckoff, Yacov Rabi

**Affiliations:** a Stanford University Medical Center, Division of Neonatology, 453 Quarry Road, Palo Alto, CA 94304, United States; b Department of Newborn Research, The Royal Women's Hospital, Parkville, VIC 3052, Australia; c Gandel Simulation Service and Department of Obstetrics & Gynaecology, The University of Melbourne, Parkville, VIC 3010, Australia; d Mater Research Institute and Mater Clinical School, Faculty of Medicine, The University of Queensland, Brisbane, Queensland, Australia; e Division of Neonatal-Perinatal Medicine, Department of Pediatrics, The University of Texas Southwestern Medical Center, Dallas, TX, United States; f University of Calgary, 1403 29 St NW, Calgary, AB T2N 2T9, Canada; g Alberta Children’s Hospital Research Institute, 28 Oki Dr NW, Calgary, AB T3B 6A8, Canada

**Keywords:** Neonatal resuscitation, Respiratory function monitoring, Grading of Recommendations, Assessment, Development and Evaluations (GRADE), Positive pressure ventilation (PPV), International Liaison Committee on Resuscitation (ILCOR), Neonatal Life Support Task Force (NLS TF), Preferred Reporting Items for Systematic Reviews and meta-analyses (PRISMA), GRADE, Grading of Recommendations, Assessment, Development and Evaluations, R F M, Respiratory Function Monitoring, RCTs, randomized controlled trials, NICU, neonatal intensive care unit, ILCOR, International Liaison Committee on Resuscitation, NLS TF, Neonatal Life Support Task Force, PRISMA, Preferred Reporting Items for Systematic Reviews and meta-analyses, ECG, electrocardiogram, CINAHL, Cumulative Index to Nursing and Allied Health Literature, RoB, risk of bias

## Abstract

**Aim:**

Positive pressure ventilation via a facemask is critical in neonatal resuscitation, but frequently results in mask leak, obstruction, and inadequate respiratory support. This systematic review aimed to determine whether the display of respiratory function monitoring improved resuscitation or clinical outcomes.

**Methods:**

Randomized controlled trials comparing outcomes when respiratory function monitoring was displayed versus not displayed for newborns requiring positive pressure ventilation at birth were selected and from databases (last search August 2022), and assessed for risk of bias using Cochrane Risk of Bias Tools for randomized control trials. The study was registered in the Prospective Register of Systematic Reviews. Grading of Recommendations, Assessment, Development and Evaluations was used to assess the certainty of evidence. Treatment recommendations were approved by the Neonatal Life Support Task Force of the International Liaison Committee on Resuscitation. Results reported primary and secondary outcomes and included resuscitation and clinical outcomes.

**Results:**

Of 2294 unique articles assessed for eligibility, three randomized controlled trials were included (observational studies excluded) (n = 443 patients). For predefined resuscitation and clinical outcomes, these studies either did not report the primary outcome (time to heart rate ≥ 100 bpm from birth), had differing reporting methods (achieving desired tidal volumes, significant mask leak) or did not find significant differences (intubation rate, air leaks, death before hospital discharge, severe intraventricular hemorrhage, chronic lung disease). Limitations included limited sample size for critical outcomes, inconsistent definitions amongst studies and unreported long-term outcomes.

**Conclusion:**

Although respiratory function monitoring has been utilized in clinical care, there is currently insufficient evidence to suggest its benefit for newborn infants receiving respiratory support for resuscitation at birth.

**Registration:**

PROSPERO CRD42021278169 (registered November 27, 2021).

**Funding:**

The International Liaison Committee on Resuscitation provided support that included access to software platforms and teleconferencing.

## Introduction

At birth, newborn infants undergo multiple physiologic changes, including lung aeration, airway liquid clearance, and the initiation of pulmonary gas exchange.^1^ Approximately 5% of term newborns need respiratory support to successfully complete this transition, whereas advanced resuscitation interventions are needed in less than 1%.^2^ Providing rapid and effective positive pressure ventilation via a face mask is considered to be a critical component of neonatal resuscitation.^3^, ^4^, ^5^ However, this is a challenging skill to master and maintain.^6^, ^7^ Frequent problems when providing mask ventilation are: a widely variable mask leak [median (range) of 29% (0%–100%]^8^, ^9^ and mask obstruction; which may lead to an inadequate tidal volume being delivered.^10^ Respiratory function monitoring may help clinicians improve resuscitation performance by providing feedback on mask leak and delivered tidal volumes, among other parameters. In randomized controlled trials (RCTs) the use of respiratory function monitoring reduces face mask leak.^11^, ^12^, ^13^, ^14^

Studies with respiratory function monitoring have demonstrated that changes in tidal volume occur during transition at birth,^15^ a positive relationship between tidal volume delivered and increase of heart rate during this transition,^16^ the contribution of spontaneous breathing to the tidal volume in newborn infants being provided positive pressure ventilation^17^, ^18^ and tidal volume changes during cardiac compressions.^19^ Clinically, respiratory function monitoring via mechanical ventilators is commonly used in the neonatal intensive care unit (NICU) as a feedback tool.^20^ However, it is not routinely used to monitor ventilation during neonatal resuscitation. Instead, the assessment of adequate ventilation in the delivery room relies on observing adequate chest rise, and heart rate improvement. T-piece resuscitator devices deliver a known peak inflation pressure and positive end expiratory pressure. However, peak inflation pressure may not correlate with delivered tidal volume, which will vary depending on face mask leak and obstruction, lung aeration, as well as lung compliance and airway resistance.^21^ Respiratory function monitoring helps identify mask leak and obstruction, and measures the expired tidal volume. Most clinicians underestimate face-mask leak, and thereby, their estimation of delivered tidal volume is poor.^22^ Respiratory function monitoring has potential to replace inaccurate and imprecise visual estimation of tidal volume by providing a more accurate data display.^23^, ^24^ The International Liaison Committee on Resuscitation (ILCOR) Neonatal Life Support Task Force (NLS TF) identified respiratory function monitoring as a high priority topic and had reviewed this topic in 2015.^25^ Literature surveillance identified new trials that justified a review update. This systematic review aimed to determine whether the display of respiratory function monitoring improved resuscitation or clinical outcomes.

## Methods

### Protocol

This study was conducted in accordance with Cochrane Handbook for Systematic Reviews of Interventions.^26^ Reporting followed the Preferred Reporting Items for Systematic Reviews and Meta-Analyses (PRISMA) statement for meta-analyses in healthcare protocol.^27^ The study was registered in the Prospective Register of Systematic Reviews (PROSPERO) (CRD42021278169, registered November 27, 2021) before beginning data extraction. This review included studies in newborn infants receiving respiratory support at birth to determine if the display of respiratory function monitoring versus no display of respiratory function monitoring improve resuscitation and/or improve clinical outcomes.

Respiratory function monitoring was defined as a device(s) that measures the following parameters during neonatal resuscitation: 1. Calculated or measured by flow meter: mask leak, inspired and expired tidal volume, flow rate, respiratory rate, 2. Measured by manometry: peak inflation pressure, positive end expiratory pressure, 3. Measured by capnography: end-tidal carbon dioxide concentration excluding colorimetric detectors (optional). As defined for this review, respiratory function monitoring does not include unintegrated stand-alone electrocardiogram (ECG), pulse oximetry or an analog display of manometry.

PROSPERO was updated following discussions with the NLS TF and our ILCOR representatives to reflect the following changes. The primary outcome of death before discharge was initially selected, but it was determined prior to the search that HR > 100 bpm was a more appropriate primary outcome, given its importance as a marker of successful resuscitation and its influence on the decisions of the health care team; and European Union trials were inadvertently left out of the registry, but the search was in fact performed and PROSPERO was amended.

### Outcomes

Published evidence and discussion with the ILCOR NLS TF was utilized for the ranking of patient-oriented outcomes.^28^ Outcomes of interest were broadly categorized into ‘resuscitation outcomes’ [time to heart rate ≥ 100 bpm from birth (primary outcome), achieving desired tidal volume, maximum mask leak, rate of intubation] and ‘clinical outcomes’ (death before hospital discharge, severe intraventricular hemorrhage (grades 3 or 4), bronchopulmonary dysplasia or chronic lung disease, duration of respiratory support, air leaks) reported either individually or as a composite outcome.

### Search strategy

A search was conducted by an information specialist in close consultation with the review team in the following databases, from their date of inception until September 20, 2021 without language restrictions: Ovid Medline, Embase, Cochrane Controlled Register of Trials, Cumulative Index to Nursing and Allied Health Literature (CINAHL), US National Library of Medicine (clinicaltrials.gov), International Standard Randomized Controlled Trial Number registry (isrctn.com) and the European Union Clinical Trials Register (clinicaltrialsregister.eu). The search was repeated on August 25, 2022. The search strategy for all databases is included in Supplement A.

### Study selection and data extraction

Covidence (Veritas Health Innovation, Melbourne, Australia) was used for study selection and data extraction. Titles and abstracts were screened by two independent reviewers (JF, YR). Disagreement during abstract screening was resolved by full text review. In the event initial consensus could not be reached, a third reviewer (MT) completed full text review with final decisions determined by team consensus.

RCTs and non-randomized studies (non-RCTs, interrupted time series, controlled before-and-after studies, cohort studies), manikin-based studies, and animal-based studies were eligible for inclusion. Although the search strategy was designed to find animal and manikin studies, an early decision was made that because there were sufficient human infant trials to provide direct evidence, animal and manikin studies were set aside for inclusion in a future review that will include training and teamwork outcomes. Unpublished studies (e.g. conference abstracts, trial protocols) were excluded. As three randomized control trials were eligible for inclusion in this review, we did not include observational studies in the formal analysis.

### Data Collection, risk of bias and certainty of evidence Assessment

Authors independently extracted details of study methodology and prespecified outcomes. Authors reached consensus for any disagreements through discussion. The pair of authors assessed risk of bias (RoB) using the Cochrane Risk of Bias Tool for RCTs (version 2). Certainty of evidence for each outcome was assessed by pairs of authors utilizing the GRADE framework.^29^ The entire team reviewed the RoB and GRADE evaluations to achieve consensus.

### Data analysis

Data analysis was conducted using Review Manager software (version 5.3, Nordic Cochrane Centre, Copenhagen, Denmark). Evidence to decision assessments utilized GRADEpro GDT software (GRADEpro Guideline Development Tool. McMaster University and Evidence Prime, 2021).

All prespecified outcomes were reported in this review, no extra data provided by study authors was requested. A meta-analysis using Revman Forest plots was performed if at least 2 studies were included for the relevant outcome. Where meta-analysis was not appropriate, but prespecified outcome was important (e.g. achieving desired tidal volume, significant mask leak), studies were included in a narrative description. Heterogeneity was quantified using the I^2^ statistic. Given our expectation for small sample sizes, we employed a random effects model. We calculated unadjusted risk ratios using the Mantel-Haenszel method for dichotomous variables. Prespecified subgroup analyses were conducted for all outcomes where data was available and included: i. gestational age at birth: ≥37 weeks, 32–36 weeks, <32 weeks, ii. timing of cord clamping: <30 seconds (immediate), ≥30 seconds (deferred).

## Results

**Fig. 1:**
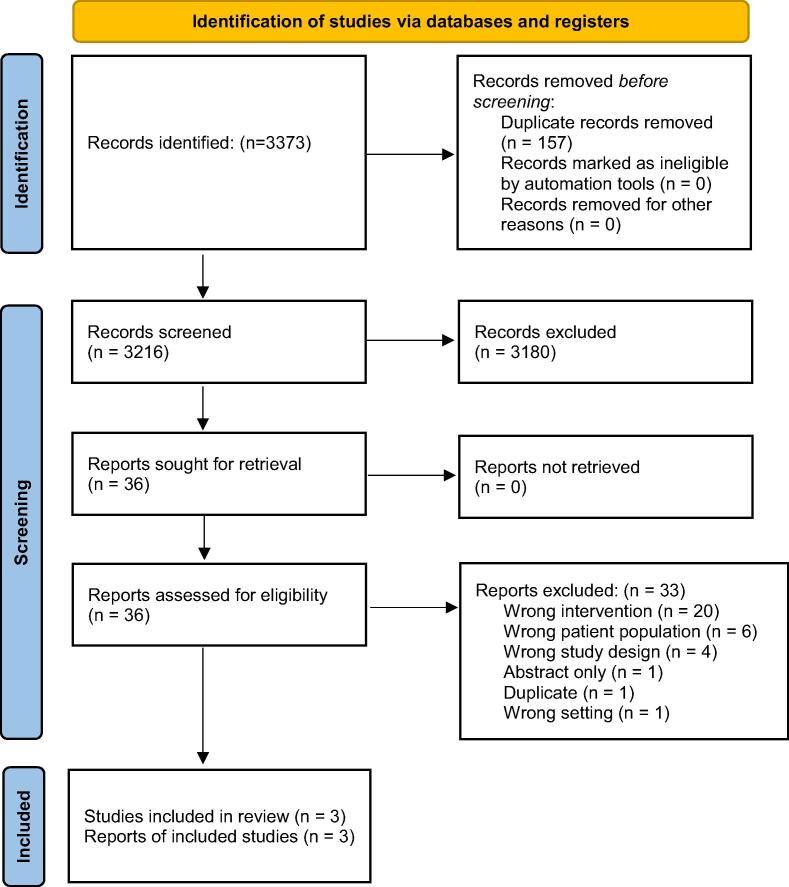
**PRISMA Flow Diagram 32** Studies were excluded because they were categorized as observational studies on humans or simulated patients that will be included in a future systematic review examining team performance. From: Page et al.^27^

Our search identified 2807 studies (513 duplicates, 2259 deemed irrelevant) with 35 full-text studies assessed for eligibility; of these, three RCTs^30^, ^31^, ^32^ were included in the final analysis and 32 observational studies were ultimately excluded, but will be included in a future systematic review examining human performance. Cohen’s kappa was 0.72 (substantial agreement) at the abstract screening stage and 1.0 (full agreement) at the full-text screening stage. Refer to the Covidence PRISMA flow diagram (Fig. 1) and the GRADE Assessment of Evidence table (Table 1).

**Table 1:** **GRADE Assessment of Evidence table** Classification criteria for secondary outcomes varied across the studies and were not consistently specified: Zeballos Sarrato - IVH (Papile classification), necrotizing enterocolitis [NEC (Bell’s staging)], no classification criteria for BPD, PVL, retinopathy of prematurity (ROP), or patent ductus arteriosus (PDA); Schmölzer - death or grade 4 IVH (Papile classification), no classification criteria for BPD, PVL, ROP, NEC or PDA^30^; van Zanten - IVH (Papile classification), BPD , no classification criteria for PVL, ROP, NEC or PDA.

Outcomes	№ of participants	Certainty of the evidence	Relative effect	Anticipated absolute effects* (95 % CI)	
	(studies) Follow-up	(GRADE)	(95 % CI)	**Risk with no respiratory function monitoring**	**Risk difference with respiratory function monitoring**
Intubation in delivery room	443	⊕○○○	**RR 0.90**	Study population	
	(3 RCTs)^1^^,^^2^^,^^3^	Very low^a^^,^^b^^,^^c^	(0.55 to 1.48)	353 per 1,000	**35 fewer per 1,000**
					(159 fewer to 169 more)
Achieving targeted TVs (4–8 mL/kg)	337	⊕⊕○○	**RR 0.96**	Study population	
	(2 RCTs)^1^^,^^3^	Lowa,d	(0.69 to 1.34)	301 per 1,000	**12 fewer per 1,000**
					(93 fewer to 102 more)
BPD	393	⊕⊕○○	**RR 0.85**	Study population	
	(2 RCTs)2,3	Lowa,e	(0.70 to 1.04)	527 per 1,000	**79 fewer per 1,000**
					(158 fewer to 21 more)
	287	⊕⊕○○	**RR 0.96**	Study population	
IVH (Grade 3 or 4)	(1 RCT)3	Low^a^^,^^e^	(0.38 to 2.42)	60 per 1,000	**2 fewer per 1,000**
					(37 fewer to 86 more)
Death prior to hospital discharge	442	⊕⊕○○	**RR 1.00**	Study population	
	(3 RCTs)^1^^,^^2^^,^^3^	Lowa,c	(0.66 to 1.52)	165 per 1,000	**0 fewer per 1,000**
					(56 fewer to 86 more)
Pneumothorax	393	⊕⊕○○	**RR 0.54**	Study population	
	(2 RCTs)2,3	Low^a^^,^^d^	(0.26 to 1.13)	95 per 1,000	**43 fewer per 1,000**
					(70 fewer to 12 more)
IVH (all grades)	393	⊕⊕○○	**RR 0.69**	Study population	
	(2 RCTs)2,3	Low^a^^,^^c^	(0.49 to 0.96)	318 per 1,000	**99 fewer per 1,000**
					(162 fewer to 13 fewer)

1 Schmölzer GM, Morley CJ,Wong C,Dawson JA,Kamlin CO,Donath SM,Hooper SB,Davis PG. Respiratory function monitor guidance of mask ventilation in the delivery room: a feasibility study. J Pediatr; 2012.

2 Zeballos Sarrato G, Sánchez Luna M,Zeballos Sarrato S,Pérez Pérez A,Pescador Chamorro I,Bellón Cano JM. New Strategies of Pulmonary Protection of Preterm Infants in the Delivery Room with the Respiratory Function Monitoring. Am J Perinatol; 2019.

3 van Zanten HA, Kuypers KLAM,van Zwet EW,van Vonderen JJ,Kamlin COF,Springer L,Lista G,Cavigioli F,Vento M,Núñez-Ramiro A,Oberthuer A,Kribs A,Kuester H,Horn S,Weinberg DD,Foglia EE,Morley CJ,Davis PG,Te Pas AB.. A multi-centre randomised controlled trial of respiratory function monitoring during stabilisation of very preterm infants at birth. Resuscitation; 2021.

a Lack of blinding for intervention; 2 studies with some concerns for selective reporting; 3 studies had high or serious concerns for overall risk of bias.

b Moderate - I^2^ = 61 %.

c Wide confidence interval.

d Wide confidence interval / Small sample size.

e Wide confidence interval, small sample size, single study, remote outcome.

### Study characteristics

Three RCTs^30^, ^31^, ^32^ were identified, including 443 newborn infants. One newborn infant died in the delivery room in the van Zanten et al. study, resulting in a total of 442 newborn infants available for analysis. The studies were conducted in Australia, Europe and North America and were published between 2012 and 2021. Patients were recruited from 2008-2019. Two studies were conducted at a single center^30^, ^31^, ^33^and the third study was multi-centered.^32^ The studies ranged in size from 49 to 288 infants. The three RCTs enrolled infants < 37 weeks postmenstrual age who required positive pressure ventilation in the delivery room and infants were randomized to an respiratory function monitoring displayed or respiratory function monitoring not displayed group (Table 4).

### Assessment of sources of bias

All three studies had potential bias regarding blinding of personnel (Table 2). Although all had concealment of the allocation sequence, there may have been team's performance bias because the intervention itself (respiratory function monitoring displayed vs not displayed) could not be blinded due to nature of the studies' design. One study (Zeballos Sarrato et al.) did not specify if outcome assessors were blinded.^31^ Furthermore, in this study, tidal volume was specified as the primary outcome in the clinical trial registry (USClinicalTrials.gov PRS, ID: NCT02748720), whereas the primary outcome reported in the published study was need for surfactant (selective reporting bias). Likewise, Schmölzer et al. listed several secondary outcomes in the clinical registry (ACTRN12608000357358) that were not ultimately reported (changes in heart rate and SpO_2_ during the first 10 minutes, days of ventilation, O_2_ at 36 weeks’ postmenstrual age).^30^ As a result, overall risk of bias was assessed as ‘high’ for Zeballos et al. and Schmölzer et al. and ‘some concerns’ for van Zanten et al.^30^, ^31^, ^32^

**Table 2:** Risk of Bias for the three RCTs evaluated.

**Manuscript**	**Random sequence generation**	**Allocation Concealment**	**Blinding of participants and personnel**	**Blinding of outcome assessment**	**Incomplete outcome data**	**Selective reporting**	**Overall Risk of Bias**
**Schmölzer et al., 2012^1^**	**Low**	**Low**	**Some Concerns**	**Low**	**Low**	**Some concern**	**High**
**Zeballos Sarrato et al., 2018^2^**	**Low**	**Low**	**Some Concerns**	**Low**	**Low**	**Some concern**	**High**
**Van Zanten et al., 2021^3^**	**Low**	**Low**	**Some Concerns**	**Low**	**Low**	**Low**	**Some Concerns**

### Primary outcomes

For the important outcome of time to heart rate > 100 bpm in the delivery room, no data were reported in the included studies.

### Secondary Outcomes:

**Fig. 2:**
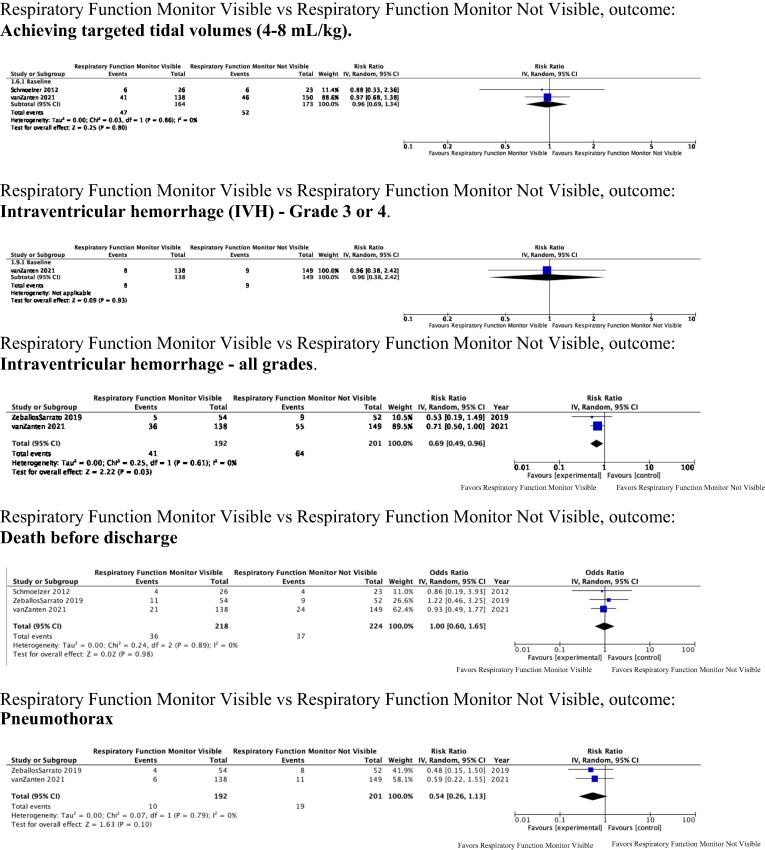
**Forest Plots:** Selected data represented here while the remaining Forest Plots are listed in Supplement C.

Forest plots are displayed in Fig. 2.

### Resuscitation outcomes

Pre-specified resuscitation outcomes for this review included: time to heart rate > 100 bpm, achieving desired tidal volume, maximum mask leak and rate of intubation. Other outcomes were considered post-hoc analyses.

For the important outcome of intubation in the delivery room, evidence of very low certainty (downgraded for risk of bias, inconsistency and imprecision) (RR 0.90, 95 % CI 0.55 – 1.48; *p* = 0.69; I^2^ = 61 %) could not exclude benefit or harm from displaying respiratory function monitoring compared to not displaying respiratory function monitoring.^30^, ^31^, ^32^

For the important outcomes of achieving desired tidal volumes in the delivery room^30^, ^32^ (RR 0.96, 95 % confidence interval (CI) 0.69 – 1.34; *p* = 0.8; I^2^ = 0 %) and, pneumothorax^31^, ^32^ (RR 0.54, 95 % CI 0.26 – 1.13; *p* = 0.10; I^2^ = 0 %), evidence of low certainty (downgraded for risk of bias and imprecision) could not exclude clinical benefit or harm from displaying respiratory function monitoring compared to not displaying respiratory function monitoring.

For the important outcome of face mask leak, the three RCTs could not be meta-analyzed as the measurement of leak was reported differently in each study. One trial reported median (IQR) mask leak per infant for the first 40 inflations and found a lower median leak when respiratory function monitoring was displayed (*p* = 0.01).^30^ Another trial reported percentage of leak > 75 % in the first 10 minutes and found less leak when respiratory function monitoring was displayed (*p* = 0.001).^31^ The third and largest trial reported median (IQR) percentage of leak > 60 % per infant also in the first 10 min and found no significant difference in leak (*p* = 0.13) between when respiratory function monitoring was displayed and not displayed.^32^

All three studies reported percentage of infants with tidal volume > 8 mL/kg, and two showed a lower proportion of infants with “excessive tidal volume” when respiratory function monitoring was displayed compared to when it was not displayed.^30^, ^31^ Schmölzer et.al.^30^ found a difference of 31 % vs 36 % of infants, (RR 0.81, 95 % CI 0.67–0.98). In a post-hoc analysis, Zeballos Sarrato et al.^31^ reported a difference of 14.8 vs 36.5 %, *p* < 0.001. However, van Zanten et.al.^32^ did not find significant differences in the percentage tidal volume > 8 mL/kg per infant (*p* = 0.93) nor the duration of tidal volume > 8 mL/kg in seconds per infant (*p* = 0.14).^32^

In regard to prespecified subgroup analyses for the systematic review, Zeballos Sarrato et al.^31^ found there was a lower proportion of infants with tidal volumes > 8 mL/kg (28–29 weeks' gestation − 25 vs 78 %, *p* < 0.001 (n = 21), <28 weeks’ gestation − 15 vs 44 %, *p* < 0.001 (n = 51).^31^ However, this was a post hoc analysis with relatively few patients and where the duration of tidal volume > 8 mL/kg was not specified, hence, it did not influence our conclusions.

Two RCTs reported on positive pressure ventilation duration using medians (IQR). Neither found a significant difference. Zeballos Sarrato et al. reported a median (IQR) positive pressure ventilation duration of 100 (63–131) seconds when respiratory function monitoring was visible and 80 seconds (45–146) when it was masked, *p* = 0.44.^31^ van Zanten et al. reported a median (IQR) positive pressure ventilation duration of 184 seconds (101–331) when respiratory function monitoring was visible and 170 seconds (82–292) when it was masked, *p* = 0.24.^32^

### Clinical outcomes

For the critical outcome of death before hospital discharge, evidence of low certainty (downgraded for risk of bias and imprecision) from 3 RCTs^30^, ^31^, ^32^ involving 442 patients could not exclude clinical benefit or harm from displaying respiratory function monitoring compared to not displaying respiratory function monitoring (RR 1.00 95 % CI 0.66 – 1.52; *p* = 0.99; I^2^ = 0 %).

For the important outcome of bronchopulmonary dysplasia /chronic lung disease (any), evidence of low certainty (downgraded for risk of bias and imprecision) from 2 RCTs^31^, ^32^ involving 393 patients could not exclude clinical benefit or harm from displaying respiratory function monitoring compared to not displaying respiratory function monitoring (RR 0.85 95 % CI 0.7 – 1.04; *p* = 0.12; I^2^ = 0 %).

For the critical outcome of severe intraventricular hemorrhage (grades 3 or 4), evidence of low certainty (downgraded for risk of bias and imprecision) from 1 RCT^32^ involving 287 patients could not exclude clinical benefit or harm from displaying respiratory function monitoring compared to not displaying respiratory function monitoring (RR 0.96 95 % CI 0.38 – 2.42; *p* = 0.93). Statistical heterogeneity could not be calculated because events occurred in only one trial.^32^

### Post-Hoc analyses

For the outcome of intraventricular hemorrhage (all grades), evidence of low certainty (downgraded for risk of bias and imprecision) from 2 RCTs^31^, ^32^ involving 393 patients suggests possible clinical benefit from displaying a respiratory function monitor compared to not displaying a respiratory function monitoring (RR 0.69 95 % CI 0.49–0.96; *p* = 0.03; I^2^ = 0 %).

Intraventricular hemorrhage (all grades) was not a pre-specified outcome for this review and should be considered a post-hoc analysis. Intraventricular hemorrhage (all grades), but not severe intraventricular hemorrhage, was significantly decreased in the respiratory function monitoring visible group (low certainty). The composite outcome of intraventricular hemorrhage (all grades) and periventricular leukomalacia was not considered for this review as the composite outcome was a post-hoc analysis and the results driven by the increased incidence of intraventricular hemorrhage (all grades), not periventricular leukomalacia which was found in only a small proportion of infants.

## Discussion

This systematic review of the use of respiratory function monitoring during neonatal resuscitation included three RCTs^30^, ^31^, ^32^ in 443 neonates (high RoB, very low or low certainty evidence) which together, did not provide support for the routine use of respiratory function monitors to guide assisted ventilation during the resuscitation of preterm infants in the delivery room.

One of the most important indicators of the effectiveness of ventilation in a newborn infant undergoing resuscitation is the response of the patient’s heart rate. Thus, it is reasonable to consider increasing heart rate as a surrogate marker for appropriate positive pressure ventilation technique, including but not limited to the use of appropriate mask size, proper mask placement, and avoidance of leak. The NLS TF determined this was the most important indicator of successful resuscitation and should be included in future respiratory function monitoring clinical studies. Unfortunately, no data on time to heart rate > 100 bpm in the delivery room were reported in the included studies. Furthermore, this review shows no significant resuscitation or clinical outcome advantages to utilizing respiratory function monitoring.

This review found low certainty evidence consistent with either clinical benefit or harm for death before discharge and is therefore consistent with a previous meta-analysis that revealed no difference in mortality.^33^ Downgrading for risk of bias and because the optimal information size for this outcome was not met by the included studies means the results are consistent with either clinical benefit or harm. The other outcome of the review that was predefined as critical, severe intraventricular hemorrhage (grades 3 and 4) was not reduced by a visible respiratory function monitoring. Reduction in intraventricular hemorrhage (all grades) could be a chance finding among the numerous reported outcomes for the included studies. Furthermore, optimal information size for these outcomes was not met by the combined included studies, contributing to the low certainty of evidence for intraventricular hemorrhage outcomes and to a judgement of only ‘possible clinical benefit.’^34^

A strong theoretical argument for respiratory function monitoring is to eliminate or reduce face mask leak during positive pressure ventilation. However, results were inconsistent with two studies^30^, ^31^ reporting less leak when respiratory function monitoring was used, whereas the third and largest study^32^ found no difference between groups. Face mask leak was measured differently in each study which precluded its meta-analysis. It would be helpful if the definition of face mask leak was standardized in future research.

Respiratory function monitoring is one of the most recent devices to be introduced to delivery rooms to assist in the care of the newborn. Those responsible for resuscitating the newborn must visualize the data that is being presented on the monitor.^35^ The optimal methods of displaying data (font style, size, color, brightness, etc.), the location of the monitor, and the types of alarms are unknown. Data must be noticed, acquired, and translated into actionable information. This complex process may be more challenging in centers where only infrequently encounter the need for resuscitation of a newborn infant.

While the literature contains simulation-based observational studies that cite potential benefits of the use of respiratory function monitoring, only a small proportion are RCTs involving human newborn infants. In general, systematic analyses conducted via ILCOR prefer RCTs conducted in human patients, and hence our analysis is based on the three human RCTs conducted to date. That said, there is an important role to be played by other research methodologies. For example, many of the human factor issues can be studied in highly controlled, simulated clinical environments during realistic simulated clinical scenarios.^36^, ^37^

While respiratory function monitoring is feasible, none of the studies examined the cost of introducing this technology. Such costs include purchasing, testing, and maintaining this technology and training. These costs may prohibit use in lower-resource settings, reducing global health equity.

There are limitations to this systematic review. The conclusions cited are based on the results of only three clinical RCTs involving a total of 443 patients, an insufficient number for some of the critical and important outcomes of the review. In some instances, key clinical outcomes were characterized by different definitions; in others, no definitions were provided at all, precluding comparisons across studies. Use of the technology was not masked, although this could not be avoided. Lastly, we are unable to report on long-term outcomes as these were not available in the included studies.

Future research priorities should include examination of human factors, methods for exploring opportunities to reduce inequity, and cost-benefit analyses. Standardized definitions of methods and outcomes in future studies would permit meta-analysis of results such as mask leak and excessive tidal volumes administered. Future research foci are found in Table 3.

**Table 3:** Examples of Future Research Priorities.

Does the use of a RFM vs no RFM during neonatal resuscitation in the delivery room result in a difference in the percentage of time spent delivering a target TV?
What is the definition of clinically significant mask leak (in terms of % leak and % of time spent with that degree of leak)?
Does the use of a RFM vs no RFM during neonatal resuscitation in the delivery room result in a faster time to a heart rate > 60 bpm (and > 100 bpm)?
What is the optimal manner to display RFM data and alarms to achieve the most accurate and timely acquisition, interpretation and translation to actionable information?
What are the training requirements to achieve and maintain competency in the acquisition and accurate interpretation of data derived from RFM during neonatal resuscitation?
What is the cost effectiveness for the use of RFM (vs no RFM) during neonatal resuscitation?

**Table 4:** **Characteristics of included RCTs –** Data largely represented as mean (SD); VTe = expired tidal volume.

	**Schmölzer et al^30^**		**Zeballos Sarrato et al^31^**		**van Zanten et al^32^**	
Location	Melbourne, Australia		Madrid, Spain		Netherlands, Australia, Germany, Spain, Italy, United States	
Study enrollment	November 2008 – January 2010		October 2014 – April 2016		October 2013 – May 2019	
Hospital Location	Delivery room		Delivery room		Delivery room	
	RFM Displayed	RFM Not Displayed (control)	RFM Displayed	RFM Not Displayed (control)	RFM Displayed	RFM Not Displayed (control)
Sample Size	n = 54	n = 46	n = 54	n = 52	n = 138	n = 150
Gestational Age	28 (2)	27 (2)	28.2 (2.7)	28.4 (2.9)	26^+2^ (25^+2^ –27^+1^)	26 + 2 (25^+4^ –27^+1^)
Birth Weight (grams)	1006 (326)	919 (324)	1133 (514)	1078 (419)	822 (187)	823 (195)
Primary outcome	Mask leak		TV during PPV		Percentage of inflations during PPV within a target range (TV 4–8 mL/kg)	
Length of analysis	First 40 breaths		First 10 minutes		First 10 minutes	
Number of inflations analyzed	1,040	920	3,329	3,934	25,432	25,920
Target TV	4–8 mL/kg		4–6 mL/kg		4–8 mL/kg	
Reported expired TV	Delivered expired TV per infant mL/kg [TV < 4 mL/kg, TV 4–8 mL/kg TV > 8 mL/kg]		Delivered VTe per infant mL/kg Patients with expired TV > 8 mL/kg		Delivered Duration of expired TV > 8 mL/kg expired TV per infant mL/kg [TV < 4 mL/kg, TV 4–8 mL/kg TV > 8 mL/kg]	
Reported face mask leak	% of leak per infant		% of leak > 75 % over all inflations		Duration of leak > 60 % per infant during PPV, AND % of leak per infant	
Type of RFM used	Florian Respiratory Function Monitor		NMS, Respiratory Profile Monitor		ALD Resuscitation Monitor	

## Conclusion

Although respiratory function monitoring has been utilized in many sites, there is currently insufficient evidence to suggest (high RoB, very low or low certainty evidence) that it would be beneficial for all newborn infants receiving respiratory support at birth. Some outcomes were meta-analyzed, but heterogeneity in the definitions of some key outcomes across studies precluded pooling results.

## Conflicts of Interest

One author (MT) participated in the van Zanten RCT’s design and protocol development, but was not involved in the execution, data analysis, data interpretation or manuscript preparation. She was excluded from bias assessment of this study. One author (YR) holds patents for pulse oximeter technology to guide oxygen titration in the delivery room. Georg Schmölzer and Peter Davis are the authors of one study.^30^ Neither was involved in selection of articles for inclusion, data extraction or analysis but both acknowledged their potential intellectual conflicts of interest and participated in the Task Force discussions.

## Appendix A Supplementary material

Supplementary data 1 PRISMA Checklist
