# Supplementary material for: Respiratory function monitoring during neonatal resuscitation: A systematic review
Source: Resusc Plus. 2022 Nov 17;12:100327. doi: 10.1016/j.resplu.2022.100327 (PMC9678959; doi:10.1016/j.resplu.2022.100327)
Supplement: Supplementary file 2 [file mmc2.docx]

**Supplement A. Search Strategy**

(infant*[ti] OR baby[ti] OR babies[ti] OR preemie*[ti] OR newborn*[ti] OR prete [illegible]
neonat*[ti] OR “infant, newborn” [mesh]) AND ("respiratory rate"[tw] OR monito [illegible]
"heart rate" [mesh] OR "respiratory rate" [mesh] OR “respiratory support” [tw] O [illegible]
monitor*” [tw] OR "Monitoring, Physiologic"[Mesh:NoExp] OR "Hemodynamic [illegible]
[mesh] OR "Respiratory Function Tests" [mesh] OR “tidal volume” [mesh] OR “e [illegible]
[tw] OR “expiratory pressure” [tw] OR “inspiratory pressure” [tw] OR “tidal volu [illegible]

**B**

## Supplement C

**Forest Plots:** Selected data represented here which are remaining Forest Plots not listed in Figure 2. **Outcome: Intubation in delivery room or first 24 hours**. **Outcome: CPAP in delivery room**. **Outcome: Bronchopulmonary dysplasia (BPD)**.

Respiratory Function Monitor Visible vs Respiratory Function Monitor Not Visible, Respiratory Function Monitor Visible vs Respiratory Function Monitor Not Visible, Respiratory Function Monitor Visible vs Respiratory Function Monitor Not Visible

Favours Respiratory Function Monitor Visible Favours Respiratory Function Monit
